# A Density-Functional Study of the Conformational Preference of Acetylcholine in the Neutral Hydrolysis

**DOI:** 10.3390/molecules25030670

**Published:** 2020-02-05

**Authors:** Rizka N. Fadilla, Febdian Rusydi, Nufida D. Aisyah, Vera Khoirunisa, Hermawan K. Dipojono, Faozan Ahmad, Mudasir Mudasir, Ira Puspitasari

**Affiliations:** 1 Research Center for Quantum Engineering Design, Faculty of Science and Technology, Universitas Airlangga, Jl. Mulyorejo, Surabaya 60115, Indonesia; fadilla.rizka13@gmail.com (R.N.F.); nufidadwiaisyah@gmail.com (N.D.A.); vera.khoirunisa@tf.itera.ac.id (V.K.); ira-p@fst.unair.ac.id (I.P.); 2 Department of Engineering Physics, Faculty of Industrial Engineering, Institut Teknologi Bandung, Bandung 40132, Indonesia; dipojono@gmail.com; 3 Department of Physics, Faculty of Science and Technology, Universitas Airlangga, Jl. Mulyorejo, Surabaya 60115, Indonesia; 4 Precision Sciences & Technology and Applied Physics, Graduate School of Engineering, Osaka University, Suita 565-0871, Japan; 5 Engineering Physics Study Program, Institut Teknologi Sumatera, Jl. Terusan Ryacudu, Lampung Selatan 35365, Indonesia; 6 Department of Physics, Faculty of Mathematics and Science, Institut Pertanian Bogor, Bogor 16680, Indonesia; faozan@apps.ipb.ac.id; 7 Department of Chemistry, Faculty of Mathematics and Science, Universitas Gadjah Mada, Yogyakarta 55281, Indonesia; mudasir@ugm.ac.id; 8 Information System Study Program, Faculty of Science and Technology, Universitas Airlangga, Jl. Mulyorejo, Surabaya 60115, Indonesia

**Keywords:** acetylcholine, conformational preference, density functional theory, neutral hydrolysis

## Abstract

Acetylcholine, which is associated with Alzheimer’s disease, is widely known to have conformers. The preference of each conformer to undergo neutral hydrolysis is yet to be considered. In this study, we employed density-functional calculations to build the conformers and investigated their preference in one-step neutral hydrolysis. The results showed the preference in ten possible hydrolysis pathways involving seven acetylcholine conformers (reactant), four transition state structures, and two choline conformers (product). Three out of the seven acetylcholine conformers predicted from the results confirmed experimental findings on the conformers stability. We suggested that two out of ten possible pathways were observed in the experimental results based on agreement in reaction energy. Eventually, this study will emphasize the importance of considering acetylcholine conformers in its hydrolysis study.

## 1. Introduction

Acetylcholine (${\mathrm{ACh}}^{+}$), the organic molecule acting as neurotransmitter in the brain, is associated with the treatment of Alzheimer’s disease (AD) [1]. AD is a progressive brain disease that slowly impairs coordination among neurons and leads to loss of body function [2]. A common explanation for AD is the cholinergic hypothesis, which states the cause as ${\mathrm{ACh}}^{+}$ depletion [3,4]. Since the role of ${\mathrm{ACh}}^{+}$ is to transmit signals among neurons [5], its depletion can disturb the signal transmission in the brain and can lead to loss of body function.

One way to treat AD is by reducing the rate of ${\mathrm{ACh}}^{+}$ neutral hydrolysis [1,6], which decomposes ${\mathrm{ACh}}^{+}$ into acetic acid (AA) and choline (${\mathrm{Ch}}^{+}$) [7]. The reaction is essential to return ${\mathrm{ACh}}^{+}$ into its resting state after being activated during the signal transmission [8]. Because it is also important to preserve sufficient ${\mathrm{ACh}}^{+}$ concentration in the brain of AD patients, reducing the rate of ${\mathrm{ACh}}^{+}$ neutral hydrolysis becomes an option to compensate for ${\mathrm{ACh}}^{+}$ depletion.

Generally, the rate of neutral hydrolysis depends on the conformers [9,10,11,12]. For example, the rate constant of bornyl acetate differs from iso-bornyl acetate acid hydrolysis up to $2.6\times 10^{4}$/min, which has been the object of conformational study [13]. Therefore, the rate of ${\mathrm{ACh}}^{+}$ neutral hydrolysis is also conformation dependent. The dependency is stronger when the reaction involves an enzyme as a catalyst [14,15]. In the ${\mathrm{ACh}}^{+}$ case, at least three conformers have been investigated to understand their stability and the interconversion among the conformers and to explore the fluorination and solvent effects on each of them [16,17,18,19,20,21,22,23,24,25,26,27]. However, to the best of our knowledge, studies of ${\mathrm{ACh}}^{+}$ conformers remain limited to its stability as an individual molecule. None have considered ${\mathrm{ACh}}^{+}$ conformers when they interact with water in a neutral hydrolysis.

In this study, we report the preference of ${\mathrm{ACh}}^{+}$ conformers in a neutral hydrolysis. We consider two important things: a one-step mechanism for the reaction model and the conformation of ${\mathrm{ACh}}^{+}$ backbone dihedral angles. Despite its simplicity, the former worked well in revealing the conformational effects in the ethyl acetate neutral hydrolysis [28]. Therefore, we can focus on the conformation in one particular transition state (TS). We use the same model for ${\mathrm{ACh}}^{+}$ neutral hydrolysis to obtain the standard enthalpy of reaction and standard Gibbs energy of activation.

## 2. Computational Methods

### 2.1. Reaction and Molecular Model

**Scheme 1:**
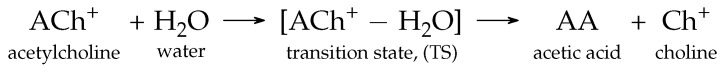
A one-step mechanism for acetylcholine neutral hydrolysis.

**Figure 1:**
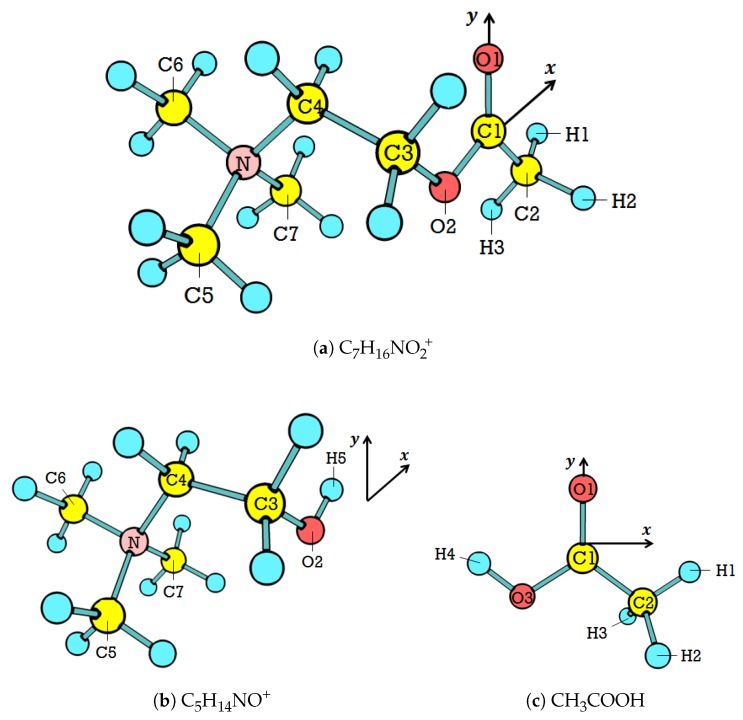
The molecular models of (**a**) ${\mathrm{ACh}}^{+}$, (**b**) ${\mathrm{Ch}}^{+}$, and (**c**) AA.

Scheme 1 represents the one-step mechanism of ${\mathrm{ACh}}^{+}$ neutral hydrolysis. Our interest is the ${\mathrm{ACh}}^{+}$ conformers because they potentially affect the activated complex in the TS and the final state (fs; products). In the TS, the activated complex is in the form of $\left[{\mathrm{ACh}}^{+}-H_{2}O\right]$. Consequently, they can affect the reaction energy and energy barrier. We assume that the initial state (is) and the fs of the reaction are infinitely separated molecules. Figure 1 shows the generic molecular models of ${\mathrm{ACh}}^{+}$, ${\mathrm{Ch}}^{+}$, and AA. Table 1 lists the geometrical parameters of interest for this study.

### 2.2. Conformer Formation

We built our initial ${\mathrm{ACh}}^{+}$ conformer based on the acetylcholine bromide (${\mathrm{ACh}}^{+}{\mathrm{Br}}^{-}$) crystal structure [29]. We removed the acetyl group ($CH_{3}CO$) from the ${\mathrm{ACh}}^{+}$ initial conformer (Figure 1a) to build our ${\mathrm{Ch}}^{+}$ initial conformer. We divided ${\mathrm{ACh}}^{+}$ into three parts, backbone (represented by D1, D2, and D3), head (represented by D4), and tail (represented by D5), and ${\mathrm{Ch}}^{+}$ into two parts, backbone (represented by D6 and D7) and tail (represented by D8), as listed in Table 1.

We varied the dihedral angles of the backbone, the head, and the tail of the initial conformer to build the potential conformers. For the ${\mathrm{ACh}}^{+}$ backbone, we varied the dihedral angles (D1, D2, and D3) with the values of $0^{{}^{\circ}}$, $-90^{{}^{\circ}}$, $+90^{{}^{\circ}}$, and $180^{{}^{\circ}}$ that yielded $4^{3}$ (four values for each of the three dihedral angles) permutations. We applied the same procedure for the ${\mathrm{Ch}}^{+}$ backbone (D6 and D7) that yielded $4^{2}$ (four values for each of two dihedral angles) permutations. For the head and the tail, we varied the dihedral angles (D4, D5, and D8) between $0^{{}^{\circ}}$ and $180^{{}^{\circ}}$, with increments of $20^{{}^{\circ}}$.

**Figure 2:**
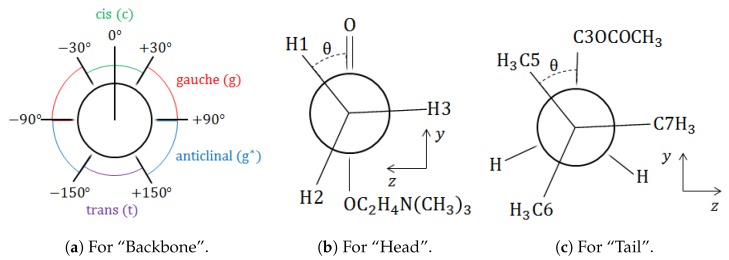
(**a**) The criteria to define the conformation of each dihedral angle. A line lying on 0° represents the bond of the first two atoms describing the dihedral angle. Newman projections (b) along C2–C1 and (c) along N–C4. For Figure 2b,c, $\theta ≅0^{{}^{\circ}}$ means eclipsed conformation and $\theta ≅60^{{}^{\circ}}$ means staggered conformation.

Figure 2 depicts the criteria for the nomenclature of stable conformers. Figure 2a shows the criteria for each of the dihedral angles constructing the backbone. For the ${\mathrm{ACh}}^{+}$ backbone, three letters representing D1, D2, and D3 describe the conformation type. The letters are written in a bracket following the corresponding conformer. For example, ${\mathrm{ACh}}^{+}$(ctg) that indicates an ${\mathrm{ACh}}^{+}$ conformer with D1, D2, and D3 are “c” (*cis*), “t” (*trans*), and “g” (*gauche*), respectively, and “g*” is for the *anticlinal* conformation. Figure 2b,c shows the criteria to define the head and the tail conformations, which can be eclipsed or staggered. We used the same nomenclature for ${\mathrm{Ch}}^{+}$ conformers.

### 2.3. Energy and Structure Calculations

We employed routines of calculations based on density functional theory (DFT) [30,31] to determine the energy and the structure of molecules in the ground state and in the TS. We used B3LYP functionals and the 6-311++G(d,p) basis set integrated in Gaussian 09 software [32]. The use of B3LYP functionals follows its success in our previous similar study on chemical reactions [28,33,34] and other similar cases [35,36]. The optimization–routine calculations are to obtain the stable structures and the total electronic energy of ${\mathrm{ACh}}^{+}{\mathrm{Br}}^{-}$, water, and AA and, more importantly, to find the stable conformers of ${\mathrm{ACh}}^{+}$ and ${\mathrm{Ch}}^{+}$. For the TS, we followed the same procedure used in our previous study [28], where we applied the TS optimization and the intrinsic reaction coordinate routines of calculation. Besides the energy and the structure, we also calculated the charge population using the Natural Bond Orbital (NBO) program [37].

### 2.4. Thermochemistry Calculations

We calculated the standard enthalpy of reaction ($\Delta_{r}H^{{}^{\circ}}$) and the standard Gibbs energy of activation ($\Delta^{‡}G^{{}^{\circ}}$) of ${\mathrm{ACh}}^{+}$ neutral hydrolysis using the following formula:

(1) $$\Delta_{r}H^{{}^{\circ}}=\left({H^{{}^{\circ}}}_{{\mathrm{ACh}}^{+}}+{H^{{}^{\circ}}}_{H_{2}O}\right)-\left({H^{{}^{\circ}}}_{\mathrm{AA}}+{H^{{}^{\circ}}}_{{\mathrm{Ch}}^{+}}\right)$$

(2) $$\Delta^{‡}G^{{}^{\circ}}=\left({G^{{}^{\circ}}}_{TS}\right)-\left({G^{{}^{\circ}}}_{{\mathrm{ACh}}^{+}}+{G^{{}^{\circ}}}_{H_{2}O}\right)$$

Both $H^{{}^{\circ}}$ and $G^{{}^{\circ}}$ in Equations (1) and (2) are temperature dependent, and we assumed the reaction occurred at room temperature (298.15 K). The values were determined from the total electronic energy of the respective systems with thermal corrections.

## 3. Results and Discussion

### 3.1. The Ground-State Structure

Table 2 presents the discrepancy in geometry between the experimental value and our calculations for ${\mathrm{ACh}}^{+}{\mathrm{Br}}^{-}$ in the ground state. The experimental values are from the crystal structure [29], which is comparable to our calculations in the gas phase. Overall, the values of $\Delta_{ba}$ are within the accuracy limit, according to Young [38]. It implies that B3LYP functional and the 6-311++G(d,p) basis set are appropriate for studying ${\mathrm{ACh}}^{+}$.

**Figure 3:**
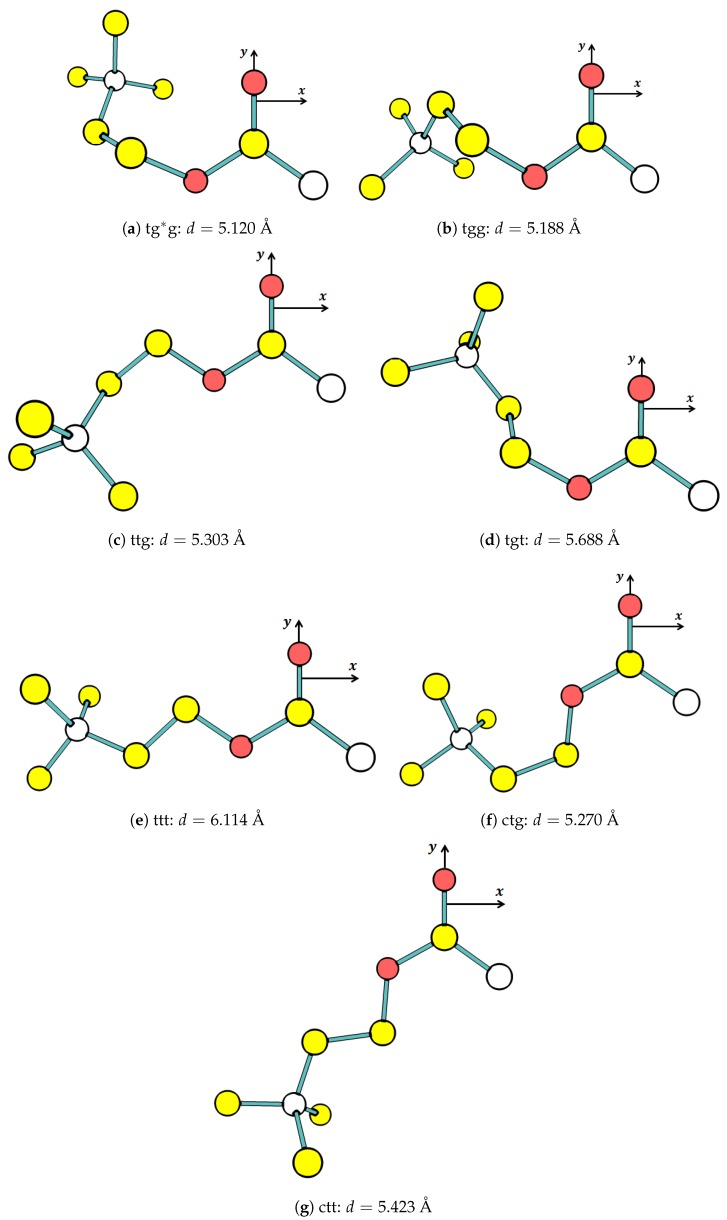
The optimized structure of ${\mathrm{ACh}}^{+}$ conformers: For clarity, all H atoms are not displayed. The distance, *d*, is between C2 and N atom (white).

The optimization routine calculations predict the stable conformer for both ${\mathrm{ACh}}^{+}$ and ${\mathrm{Ch}}^{+}$. The cartesian coordinates of the stable conformers are given in the Supplementary Materials. Only seven out of 64 potential ${\mathrm{ACh}}^{+}$ conformers are stable in the ground state, as shown in Figure 3. For ${\mathrm{Ch}}^{+}$, there are only two possible out of 16 potential conformers. Table 3 and Table 4 resume the results for ${\mathrm{ACh}}^{+}$ and ${\mathrm{Ch}}^{+}$, respectively. In both ${\mathrm{ACh}}^{+}$ and ${\mathrm{Ch}}^{+}$, the spans of the dihedral angle are more significant than those of bond lengths and bond angles, which is as expected. That is to say that the backbone determines the conformation, whereas the head (for ${\mathrm{ACh}}^{+}$ only) is always eclipsed and the tail is always staggered.

**Figure 4:**
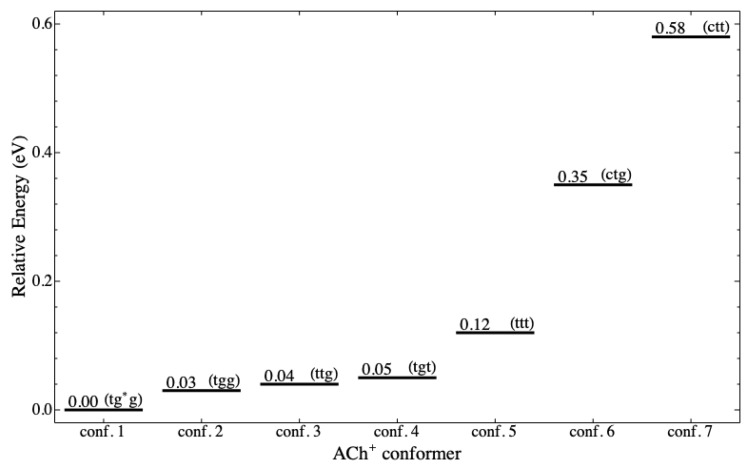
The energy level diagram (ELD) of the seven stable ${\mathrm{ACh}}^{+}$ conformers. The energy is relative to ${\mathrm{ACh}}^{+}$ (tg*g).

Figure 4 shows the energy level diagram (ELD) for the seven stable ${\mathrm{ACh}}^{+}$ conformers in eV (1 eV ≈ 23.06 kcal/mol). It is clear from the energy level that there are two groups of conformers, which are low and high level. The low-level group is more stable than the high-level group. The five ${\mathrm{ACh}}^{+}$ conformers (tg*g, tgg, ttg, tgt, and ttt) are in the low-level group (Figure 3a–e), and the other two conformers (ctg and ctt) are in the high-level group (Figure 3f,g). Other computational studies [20,23,39,40] also conclude the stability of the five low-level conformers. It is important to note that our results support the experiments that observed ${\mathrm{ACh}}^{+}$(tg*g), (tgg), and (ttg) in their stable states [29,41,42].

The ELD displays three noticeable patterns of the conformation related to ${\mathrm{ACh}}^{+}$ stability as individual molecules. The first is that *gauche* conformation at D1 cannot achieve stability, whereas *trans* and *cis* can. The second is that *cis* at D2 and D3 cannot achieve the stability, whereas *trans* and *gauche* (and *anticlinal*) can. The second pattern is as expected because the *cis* conformation causes two bulky groups (acetyl and trimethylamine) to be ecliptic, leading to a repulsive interaction among atoms of the two groups. The third is that *gauche* conformation at D2 and D3 makes ${\mathrm{ACh}}^{+}$ more stable than when they are *trans*; therefore, at the same D1, it is possible to arrange the order of ${\mathrm{ACh}}^{+}$ stability (based on D2 and D3), from the most to the least stable, as gg, tg, gt, and tt. In addition to the third pattern, it appears that the ${\mathrm{ACh}}^{+}$ stability is more dependent on D3 than D2.

Charge distributions align with this pattern. The overall NBO calculations determine that more electrons are distributed in the head, resulting in the tail being positively charged (see Table 5). It agrees with the typical ${\mathrm{ACh}}^{+}$ structure [43]. The shorter the head–tail distance, the stronger the coulombic interaction and, consequently, the more stable the conformer. Therefore, the backbone and the tail must curl up in order to shorten the head–tail distance. Such curling behavior does not only exist in gas phase but also in solvent [18,20,23,27]. The *gauche* conformation at D2 and D3 meets the condition, particularly at D3, where the head–tail distance is the shortest. Figure 3 depicts the circumstance, in which the distance gradually increases from the shortest in the g*g conformation to the longest in the tt conformation for the low-level group and from the shortest tg to the longest tt for the high-level group.

Additionally, the charge distribution shown in Table 5 indicates an electrophilic site of all ${\mathrm{ACh}}^{+}$ stable conformers. It is in the backbone, where C1 is located. This site is typical for the ester family. According to our study on ethyl acetate neutral hydrolysis [28], the activated complex (${\mathrm{ACh}}^{+}$–water) forms between C1 and O3, the nucleophilic site of water.

The electrophilic site of the ${\mathrm{ACh}}^{+}$ conformers gives a hint to the cleaving location during the hydrolysis. The cleaving location shall be the C1–O2 bond. Therefore, we extracted the C1–O2 bonding atomic orbital from the NBO calculations as listed in Table 6. All conformers have an average bonding orbital of 0.5464 C$\left(sp^{2.90}\right)$ + 0.8375 O$\left(sp^{2.19}\right)$. This bonding is relatively weaker than the C1–O2 bond of ethyl acetate, which is 0.5898 C$\left(sp^{1.91}\right)$ + 0.8076 O$\left(sp^{1.42}\right)$ [28]. It suggests that the neutral hydrolysis of ${\mathrm{ACh}}^{+}$ is easier than that of ethyl acetate.

### 3.2. The Transition State Structure

**Figure 5:**
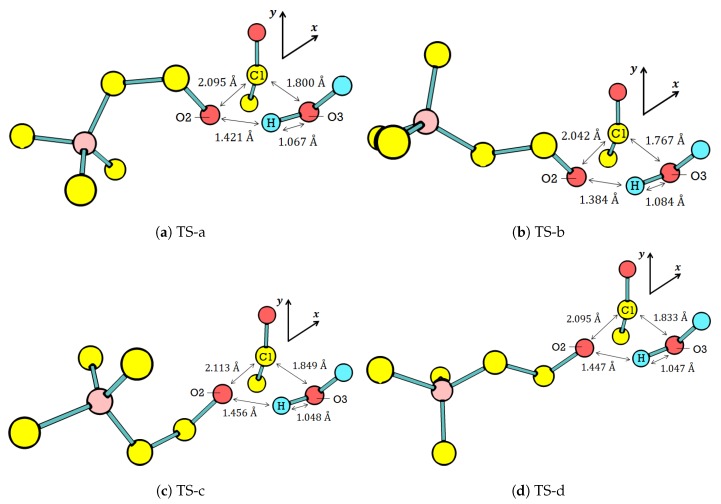
The four possible TS geometries.

The calculations narrow down the TS geometry from seven possible reactants to four [${\mathrm{ACh}}^{+}$–water] activated complexes. The cartesian coordinates of the four activated complexes are given in the Supplementary Materials. Table 7 lists the seven possible reactants (codes Re1–Re7). Figure 5 displays the optimized activated complex of these four [${\mathrm{ACh}}^{+}$–water]. The overall orientation of water with respect to ${\mathrm{ACh}}^{+}$ is similar to our previous study on [ethyl acetate–water] activated complex [28]. This similarity suggests that ${\mathrm{ACh}}^{+}$ neutral hydrolysis resembles base-induced ester hydrolysis.

The ${\mathrm{ACh}}^{+}$–water interaction in all four possible TSs elongates C1–O2, which makes it an important parameter since it is the cleaving location, as we have discussed in Table 6. The elongation of C1–O2 is around 50% (from 1.40 Å (Table 3) to 2.10 Å (Figure 5)). It is significantly larger than that of the C1–O2 in ethyl acetate–water interaction, which is around 33% [28]. The large C1–O2 elongation is explainable according to the bonding orbital of C1–O2 described in Table 6. The bond in ${\mathrm{ACh}}^{+}$ is weaker than that in ethyl acetate; therefore, the bond is easier to break in ${\mathrm{ACh}}^{+}$ relative to ethyl acetate. Consequently, ${\mathrm{ACh}}^{+}$ neutral hydrolysis is expected be faster than that of ethyl acetate. This expectation agrees with the experimental data showing that, at room temperature, the rate constant of the former is $10^{-9}$/s [44], whereas that of the latter is $10^{-10}$/s [45].

In addition to the C1–O2 elongation, there are two other similarities among the four TS geometries. First, the elongation is large enough to split the acetyl group from the rest of ${\mathrm{ACh}}^{+}$. For comparison, the generalization of C–O bond lengths in saturated molecules, like ${\mathrm{ACh}}^{+}$, has been widely assumed as 1.43 Å. Meanwhile, the O3 of water is still too far from C1 to form a covalent bond. The activated complex thus consists of three groups: water, acetyl, and choline. The three groups interact with each other through noncovalent interactions to form the activated complex, which lies on the TS. Second, ${\mathrm{ACh}}^{+}$ prefers the curling D2 and D3 in the presence of water. The curling D2 and D3 relates the ${\mathrm{ACh}}^{+}$ in the TS to the one in the ground state: (ttg), (tgt), (ctg), and (ctt). Since the curling, D2 and D3 also affect the C2–N distance and the seven ${\mathrm{ACh}}^{+}$ conformers are grouped into three curling levels. The levels are extreme (d < 5.20 Å), medium (5.30 < d < 5.70 Å), and low (d > 5.70 Å). Accordingly, all TS geometries require the medium curling level of ${\mathrm{ACh}}^{+}$ conformers.

Among the four TS geometries, TS-b is the most favorable one for product formation. Generally, the product formation requires the elongation of C1–O2 and O3–H in the TS with respect to the ground state, as well as shortening the distances of C1–O3 and O2–H (distances between groups in the activated complex). The shortened C1–O3 and O2–H promote the formation of AA and ${\mathrm{Ch}}^{+}$, respectively. TS-b meets most of the requirements for product formation as its O3–H is the longest, whereas its C1–O3 and O2–H are the shortest among the four TS geometries.

### 3.3. The Reaction Coordinate

**Figure 6:**
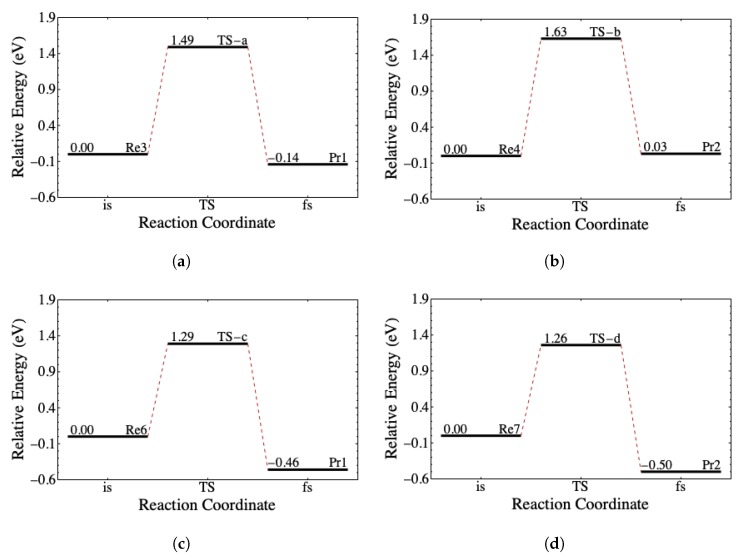
The ELDs for four possible TSs: (**a**) TS-a, (**b**) TS-b, (**c**) TS-c, and (**d**) TS-d, and their related initial (is) and final states (fs). The code of the reactants and the products follows Table 7. The relative energy of each TS corresponds to the energy barrier, whereas the relative energy of the fs corresponds to the reaction energy.

Figure 6 shows the neutral hydrolysis reaction coordinate in the ELD. The ELD involves four out of the seven potential reactants (see Table 7) capable of forming the activated complex at the TS through a one-step mechanism. The possible reactants are Re3, Re4, Re6, and Re7, which are related to the aforementioned ${\mathrm{ACh}}^{+}$ curling levels. The possible products are Pr1 and Pr2, which comprise ${\mathrm{Ch}}^{+}$(tg) and ${\mathrm{Ch}}^{+}$(tt) from Table 4. Although the TS depends on the curling levels of D2 and D3 of the ${\mathrm{ACh}}^{+}$ conformers, the products depend only on D3. Since D3 does not contain the electrophilic site, it does not change when ${\mathrm{ACh}}^{+}$ is hydrolyzed into ${\mathrm{Ch}}^{+}$.

**Figure 7:**
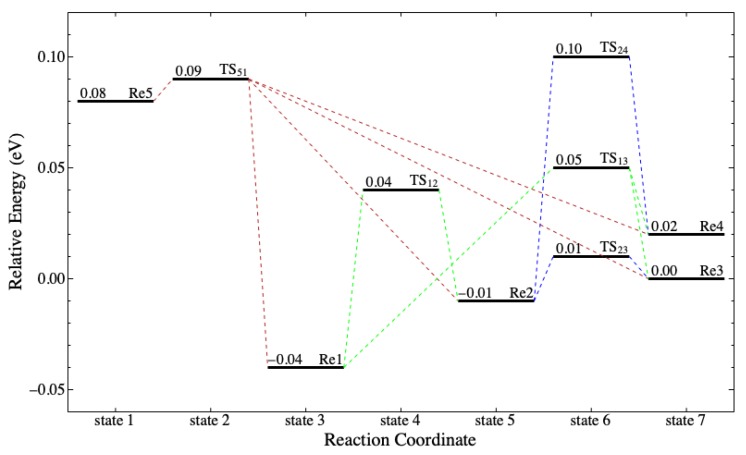
The reaction coordinates for all potential reactants in Table 7 before forming the activated complex (TS-a, TS-b, TS-c, and TS-d): TS_12_ means the transition state of conformational isomerization from Re1 to Re2.

Figure 7 shows the pre-hydrolysis reaction coordinate in the ELD. There are three out of seven potential reactants that require a pre-hydrolysis process (Re1, Re2, and Re5). These reactants need to undergo conformational isomerization to form either ${\mathrm{ACh}}^{+}$(ttg) or (tgt) with the energy barriers at no more than 0.11 eV. It implies that the conformational isomerization can occur by thermal energy. Together with Figure 6, Figure 7 suggests that all seven potential reactants can perform hydrolysis in four pathways. The reactants with the low-level group of ${\mathrm{ACh}}^{+}$ conformers go through Re3 or Re4 before going to either TS-a or TS-b. Both pathways are possible because the energy barrier to form Re3 and Re4 is no more than 0.11 eV. Meanwhile, the reactants with the high-level group of ${\mathrm{ACh}}^{+}$ conformers go directly to TS-c and TS-d.

Table 8 shows the $\Delta_{r}H^{{}^{\circ}}$ for all possible reaction coordinates in Figure 6 and Figure 7. The calculations of $\Delta_{r}H^{{}^{\circ}}$ suggest that reactants with the high-level ${\mathrm{ACh}}^{+}$ conformers are always exothermic and go toward either Pr1 or Pr2. Meanwhile, reactants from the low-level group are exothermic if they go toward Pr1, but they are endothermic if they go toward Pr2. Experimentally, the reaction is endothermic, with $\Delta_{r}H^{{}^{\circ}}$ being +0.28 kcal/mol [46]. According to our results, Pr2 is mostly the product of the hydrolysis. In particular, the experiment observed mostly reactions (viii) or (ix), suggesting that the practically preferred ${\mathrm{ACh}}^{+}$ conformer undergoes neutral hydrolysis, which is (tgt) or (ttt). It is worthwhile to mention that our results are in line with the study of Zhorov et al. [47], which suggest that ${\mathrm{ACh}}^{+}$ with D2 and D3 being *trans* is productive for the ${\mathrm{ACh}}^{+}$ hydrolysis catalyzed by acetylcholinesterase (AChE), as well as the study of Chothia and Pauling [48], which suggests that the ${\mathrm{ACh}}^{+}$ conformation relevant for its interaction with AChE is the one with D1, D2, and D3 being *trans*.

**Figure 8:**
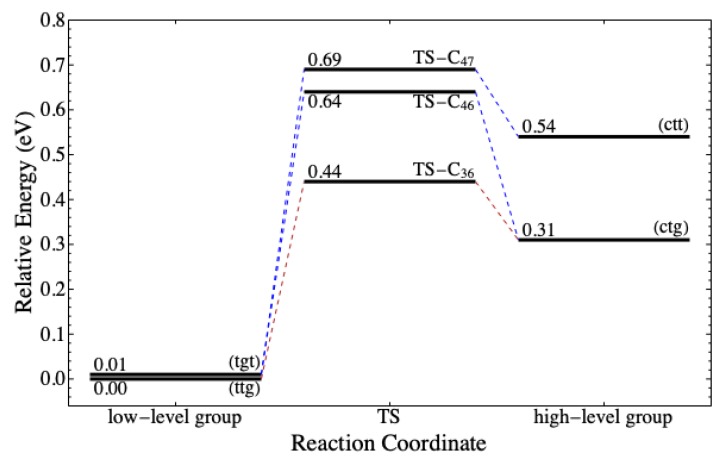
The reaction coordinates from low- to high-level groups of ${\mathrm{ACh}}^{+}$ conformers: The TS between ${\mathrm{ACh}}^{+}$(ttg) and (ctt) is almost 3 eV, and it is not displayed for the sake of clarity.

In addition to $\Delta_{r}H^{{}^{\circ}}$, Table 8 shows $\Delta^{‡}G^{{}^{\circ}}$. As expected, $\Delta^{‡}G^{{}^{\circ}}$ of reactants with the high-level ${\mathrm{ACh}}^{+}$ conformers are lower than that of reactants with the low-level conformers. Consequently, reactions (v) and (x) are favorable to occur due to the low activation energy and the high exothermicity. However, the energy level of both ${\mathrm{ACh}}^{+}$(ctt) and (ctg) are more than 0.30 eV higher than the most stable conformer. They can transform to ${\mathrm{ACh}}^{+}$(tgt) and (ttg) via conformational isomerization according to the reaction coordinate depicted in Figure 8. The energy barrier is no more than 0.33 eV, which is still in the order of thermal energy. It implies that, despite a low $\Delta^{‡}G^{{}^{\circ}}$, the number of ${\mathrm{ACh}}^{+}$(ctt) and (ctg) in nature is likely lower than that in the low-level group.

## 4. Conclusions

We have reported that each ${\mathrm{ACh}}^{+}$ conformer exhibited different conformational preferences when existing as an individual molecule and as an activated [${\mathrm{ACh}}^{+}$–water] complex of a neutral hydrolysis. As an individual molecule, we obtained seven possible ${\mathrm{ACh}}^{+}$ conformers: five low-level and two high-level conformers, each with a unique backbone conformation. Three out of the five low-level conformers were observed in the experiments. However, only four out of the seven conformers were capable of undergoing direct neutral hydrolysis via four distinct TSs, while the others had to go through some possible pre-hydrolysis pathways before forming the TS. Among the four TS structures, TS-b was the most favorable one to form the product of the neutral hydrolysis. The structure offered an insight for constructing the starting TS structure of ${\mathrm{ACh}}^{+}$ neutral hydrolysis catalyzed by AChE.

In this study, we proposed ten possible reaction pathways of ${\mathrm{ACh}}^{+}$ neutral hydrolysis. The most favorable reactions involved the high-level conformer with $\Delta^{‡}G^{{}^{\circ}}$ being 38.95 kcal/mol and $\Delta_{r}H^{{}^{\circ}}$ being −10.33 kcal/mol. Importantly, we suggested two possible reactions involving low-level conformers ((*trans, gauche, trans*) and (*trans, trans, trans*)) with $\Delta_{r}H^{{}^{\circ}}$ values of +1.38 and +0.10 kcal/mol, agreeing with the experimental observations. Furthermore, we argued that one had to consider ${\mathrm{ACh}}^{+}$ conformers when studying its hydrolysis.

Rizka N. Fadilla, Febdian Rusydi, Nufida D. Aisyah, Vera Khoirunisa, Hermawan K. Dipojono, Faozan Ahmad, Mudasir, and Ira Puspitasari

## Figures and Tables

**Table 1:** The geometrical parameters of interest from Figure 1 and the corresponding notations used throughout the manuscript.

Parameter	Definition		Unit
(a) ${\mathrm{ACh}}^{+}$			
D1	dihedral angle of C2–C1–O2–C3	(backbone)	deg.
D2	dihedral angle of C1–O2–C3–C4	(backbone)	deg.
D3	dihedral angle of O2–C3–C4–N	(backbone)	deg.
D4	dihedral angle of O1–C1–C2–H1	(head)	deg.
D5	dihedral angle of C3–C4–N–C5	(tail)	deg.
(b) ${\mathrm{Ch}}^{+}$			
D6	dihedral angle of H5–O2–C3–C4	(backbone)	deg.
D7	dihedral angle of O2–C3–C4–N	(backbone)	deg.
D8	dihedral angle of C3–C4–N–C5	(tail)	deg.

**Table 2:** The optimized geometrical parameters of ${\mathrm{ACh}}^{+}{\mathrm{Br}}^{-}$ from (a) experimental values [29] and (b) our calculations (R (in Å); A (in deg.)). The discrepancy $\Delta_{ba}$ is the value of (b) minus (a).

Parameter	(a)	(b)	$\Delta_{\mathrm{ba}}$
*R*(C1,O1)	1.192	1.202	0.010
*R*(C1,C2)	1.487	1.496	0.009
*R*(C1,O2)	1.358	1.381	0.023
*R*(O2,C3)	1.452	1.431	−0.021
*R*(C3,C4)	1.500	1.521	0.021
*R*(C4,N)	1.513	1.532	0.019
*A*(O1,C1,C2)	125.9	126.9	1.0
*A*(O1,C1,O2)	122.8	122.3	−0.5
*A*(C2,C1,O2)	111.3	110.8	−0.5
*A*(C1,O2,C3)	115.7	116.5	0.8
*A*(O2,C3,C4)	111.6	111.1	−0.5
*A*(C3,C4,N)	116.4	116.4	0.0

**Table 3:** The optimized conformation type and geometrical parameters of the stable ${\mathrm{ACh}}^{+}$ conformers (*R* (in Å); *A* and *D* (in deg.)).

Backbone Conformation	Parameters							
	R1	R2	R3	A1	A2	D1	D2	D3
tg*g	1.497	1 369	1.532	111.5	121.8	166.6	110.4	−79.7
tgg	1.498	1.383	1.531	111.4	121.4	170.6	81.7	67.0
ttg	1.499	1.389	1.533	111.2	121.1	−178.4	166.0	65.9
tgt	1.497	1.374	1.525	111.5	121.3	174.9	80.7	−157.2
ttt	1.498	1.384	1.525	111.1	121.1	180.0	180.0	180.0
ctg	1.505	1.400	1.533	117.4	116.4	−7.4	166.7	56.7
ctt	1.507	1.397	1.523	117.3	116.8	0.0	180.0	180.0
span	0.011	0.031	0.010	6.3	5.5	180.0	99.3	124.3

R1 C2–C1; R2 C1–O2; R3 C4–N; A1 C2–C1–O2; A2 O1–C1–O2.

**Table 4:** The optimized conformation type and geometrical parameters of the stable ${\mathrm{Ch}}^{+}$ conformers (*R* (in Å); *A* and *D* (in deg.)).

Backbone Conformation	Parameters						
	R4	R5	R6	A3	A4	D6	D7
tg	1.419	1.521	1.533	110.2	109.6	167.7	58.3
tt	1.414	1.530	1.522	110.3	102.6	179.5	178.4
span	0.004	0.009	0.011	0.1	7.0	11.8	121.2

R4 O2–C3; R5 C3–C4; R6 C4–N; A3 H5–O2–C3; A4 C3–C4–N.

**Table 5:** The atomic charge populations (in unit e) of ${\mathrm{ACh}}^{+}$ and water: Not available values are indicated by “n.a.”.

Molecules	Head		Backbone				Tail		
	O1	C2$H_{3}$	C1	O2	C3	C4	N	3($CH_{3}$)	O3
(a) ${\mathrm{ACh}}^{+}$								
tg*g	−0.61	0.06	0.82	−0.57	−0.07	−0.18	−0.36	1.00	n.a.
tgg	−0.58	0.05	0.81	−0.59	−0.07	−0.19	−0.35	1.00	n.a.
ttg	−0.55	0.04	0.82	−0.61	−0.07	−0.17	−0.34	1.00	n.a.
tgt	−0.55	0.06	0.82	−0.61	−0.07	−0.17	−0.35	1.00	n.a.
ttt	−0.56	0.05	0.82	−0.58	−0.06	−0.17	−0.35	1.00	n.a.
ctg	−0.52	0.02	0.82	−0.62	−0.05	−0.17	−0.35	1.01	n.a.
ctt	−0.51	0.01	0.81	−0.59	−0.05	−0.17	−0.35	1.00	n.a.
(b) Water	n.a.	n.a.	n.a.	n.a.	n.a.	n.a.	n.a.	n.a.	−0.91

**Table 6:** The Natural Bond Orbital (NBO) calculation for the C1–O2 bonding based on the linear combination of atomic orbitals $a\;C1\left(sp^{n}\right)+b\;O2\left(sp^{m}\right)$.

Molecules	C1		O2	
	a	${\mathrm{sp}}^{n}$	b	${\mathrm{sp}}^{m}$
(1) ${\mathrm{ACh}}^{+}$				
tg*g	0.5507	2.82	0.8347	2.11
tgg	0.5481	2.87	0.8364	2.20
ttg	0.5444	2.91	0.8388	2.24
tgt	0.5503	2.84	0.8350	2.19
ttt	0.5455	2.90	0.8381	2.24
ctg	0.5419	3.01	0.8404	2.17
ctt	0.5436	2.98	0.8393	2.21
average	0.5464	2.90	0.8375	2.19
(2) Ethyl acetate				
trans	0.5901	1.91	0.8073	1.42
gauche	0.5895	1.91	0.8078	1.41
average	0.5898	1.91	0.8076	1.42

**Table 7:** The code for reactants (initial state (is)) and products (final state (fs)) from Scheme 1.

Code	Systems	State
Re1	${\mathrm{ACh}}^{+}$(tg*g) + water	is
Re2	${\mathrm{ACh}}^{+}$(tgg) + water	is
Re3	${\mathrm{ACh}}^{+}$(ttg) + water	is
Re4	${\mathrm{ACh}}^{+}$(tgt) + water	is
Re5	${\mathrm{ACh}}^{+}$(ttt) + water	is
Re6	${\mathrm{ACh}}^{+}$(ctg) + water	is
Re7	${\mathrm{ACh}}^{+}$(ctt) + water	is
Pr1	AA + ${\mathrm{Ch}}^{+}$(tg)	fs
Pr2	AA + ${\mathrm{Ch}}^{+}$(tt)	fs

**Table 8:** The standard enthalpy of reaction ($\Delta_{r}H^{{}^{\circ}}$) and the standard Gibbs energy of activation ($\Delta^{‡}G^{{}^{\circ}}$) at 298.15 K (in kcal/mol): For Re5, only the shortest pathway is listed.

Number	Reaction	$\Delta_{r}H^{{}^{\circ}}$	$\Delta^{‡}G^{{}^{\circ}}$
a) Reactions that yield Pr1			
(i)	Re1 → Re2 → Re3 → Ts-a → Pr1	−1.67	45.28
(ii)	Re2 → Re3 → Ts-a → Pr1	−2.14	45.28
(iii)	Re3 → Ts-a → Pr1	−2.33	45.28
(iv)	Re5 → Re3 → Ts-a → Pr1	−4.17	45.28
(v)	Re6 → Ts-c → Pr1	−9.45	39.28
b) Reactions that yield Pr2			
(vi)	Re1 → Re4 → Ts-b → Pr2	+2.61	47.04
(vii)	Re2 → Re4 → Ts-b → Pr2	+2.14	47.04
(viii)	Re4 → Ts-b → Pr2	+1.38	47.04
(ix)	Re5 → Re4 → Ts-b → Pr2	+0.10	47.04
(x)	Re7 → Ts-d → Pr2	−10.33	38.95

## Supplementary Materials

Supplementary Materials are provided. The supplementary materials are available online.

## Abbreviations

The following abbreviations are used in this manuscript:

AA	Acetic Acid
${\mathrm{ACh}}^{+}$	Acetylcholine
${\mathrm{Ch}}^{+}$	Choline
ELD	Energy Level Diagram
TS	Transition State
