# Supplementary material for: A Density-Functional Study of the Conformational Preference of Acetylcholine in the Neutral Hydrolysis
Source: Molecules. 2020 Feb 5;25(3):670. doi: 10.3390/molecules25030670 (PMC7037165; doi:10.3390/molecules25030670)
Supplement: Supplementary file 1 [file molecules-25-00670-s001.pdf]

SUPPLEMENTARY MATERIALS

A Density-functional Study of the Conformational Preference of Acetylcholine in the Neutral Hydrolysis

Cartesian Coordinate of the Ground State Structure

1. ACh^{+} (tg*g)

Atom	x (Å)	y (Å)	z (Å)
C	-2.127641000	-0.187095000	0.112790000
O	-1.680417000	-0.917782000	0.964003000
O	-1.462816000	0.938465000	-0.294059000
C	-3.418607000	-0.359562000	-0.625040000
C	-0.370475000	1.395319000	0.514644000
C	1.000039000	1.227373000	-0.130102000
N	1.670396000	-0.150149000	-0.102272000
C	0.995419000	-1.120091000	-1.041635000
C	3.094856000	0.042069000	-0.562211000
C	1.676510000	-0.730321000	1.291304000
H	-3.304140000	-0.107073000	-1.679890000
H	-4.156431000	0.329173000	-0.202603000
H	-3.777267000	-1.379462000	-0.504400000
H	-0.426140000	0.950179000	1.507011000
H	-0.516517000	2.473242000	0.612148000
H	1.689042000	1.892168000	0.394337000
H	0.965570000	1.529270000	-1.177919000
H	0.931731000	-0.668323000	-2.029973000
H	1.597989000	-2.026041000	-1.085493000
H	0.009606000	-1.359506000	-0.657421000
H	3.608818000	0.702490000	0.134076000
H	3.588448000	-0.927753000	-0.585627000
H	3.089850000	0.480303000	-1.559113000
H	2.132488000	-0.010685000	1.970223000
H	0.653573000	-0.952718000	1.587101000
H	2.265866000	-1.645838000	1.275400000

2. ACh^+ (tgg)

Atom	x (Å)	y (Å)	z (Å)
C	-2.310096000	-0.065278000	-0.152241000
O	-2.305184000	-0.950224000	-0.964509000
O	-1.298993000	0.059225000	0.782890000
C	-3.346590000	1.002526000	0.019179000
C	-0.367860000	-1.027686000	0.842926000
C	0.732922000	-0.980030000	-0.210574000
N	1.767994000	0.143035000	-0.099456000
C	2.829252000	-0.115499000	-1.141044000
C	1.145039000	1.491154000	-0.371684000
C	2.416837000	0.158964000	1.259628000
H	-2.889756000	1.969768000	0.232571000
H	-3.979012000	0.743468000	0.873389000
H	-3.964292000	1.056116000	-0.874714000
H	-0.892629000	-1.971831000	0.680541000
H	0.022658000	-1.024731000	1.859885000
H	0.297247000	-0.903750000	-1.206225000
H	1.299757000	-1.911538000	-0.153544000
H	2.358072000	-0.158558000	-2.121514000
H	3.554069000	0.696309000	-1.111713000
H	3.320885000	-1.061773000	-0.921738000
H	0.378101000	1.685216000	0.372285000
H	1.927884000	2.246675000	-0.323666000
H	0.704212000	1.476482000	-1.367225000
H	1.683069000	0.443909000	2.009499000
H	2.814555000	-0.831630000	1.476152000
H	3.225625000	0.887730000	1.248016000

3. ACh^+ (ttg)

Atom	x (Å)	y (Å)	z (Å)
C	-2.542771000	-0.042441000	0.024713000
O	-3.001988000	-1.133886000	0.200528000
O	-1.181552000	0.138209000	-0.186942000
C	-3.276048000	1.264578000	-0.010320000
C	-0.414347000	-1.070513000	-0.202904000
C	0.964904000	-0.802218000	-0.772655000
N	1.899235000	0.109324000	0.031460000
C	3.283472000	-0.028383000	-0.553181000
C	1.475015000	1.556127000	-0.077637000
C	1.941735000	-0.291084000	1.482478000
H	-3.159789000	1.731962000	-0.991064000
H	-2.869150000	1.950744000	0.735925000
H	-4.330431000	1.087738000	0.188801000
H	-0.890505000	-1.807983000	-0.854927000
H	-0.382878000	-1.513031000	0.795433000
H	0.890702000	-0.354682000	-1.764445000
H	1.485234000	-1.757485000	-0.862191000
H	3.243379000	0.209371000	-1.614898000
H	3.950343000	0.664677000	-0.043242000
H	3.630291000	-1.050661000	-0.412213000
H	0.464554000	1.657860000	0.304936000
H	2.174933000	2.161149000	0.496725000
H	1.503821000	1.848403000	-1.125991000
H	0.975520000	-0.095023000	1.940651000
H	2.187360000	-1.349768000	1.554729000
H	2.708528000	0.300486000	1.979890000

4. ACh^+ (tgt)

Atom	x (Å)	y (Å)	z (Å)
C	2.379272000	0.216860000	0.045065000
O	1.802358000	1.192242000	0.458069000
O	1.741048000	-0.994963000	-0.067372000
C	3.808122000	0.126009000	-0.390706000
C	0.403484000	-1.034167000	0.433919000
C	-0.547592000	-0.442375000	-0.619842000
N	-1.870884000	0.086014000	-0.076460000
C	-2.723947000	0.491814000	-1.253175000
C	-2.593316000	-0.980776000	0.704720000
C	-1.651020000	1.301454000	0.795227000
H	3.884583000	-0.379861000	-1.354686000
H	4.367499000	-0.470197000	0.335383000
H	4.235814000	1.124386000	-0.446395000
H	0.356536000	-0.487099000	1.376001000
H	0.198703000	-2.088288000	0.613286000
H	-0.799035000	-1.189574000	-1.373191000
H	-0.068758000	0.403306000	-1.109362000
H	-2.908232000	-0.381915000	-1.875693000
H	-3.666258000	0.891064000	-0.882069000
H	-2.196298000	1.253821000	-1.824115000
H	-2.009224000	-1.246045000	1.583223000
H	-3.561311000	-0.591020000	1.015358000
H	-2.731959000	-1.853088000	0.067402000
H	-1.072322000	1.027204000	1.672274000
H	-1.106472000	2.049621000	0.223322000
H	-2.624340000	1.684451000	1.097657000

5. ACh^+ (ttt)

Atom	x (Å)	y (Å)	z (Å)
C	2.790858000	0.136130000	-0.000009000
O	2.829630000	1.334802000	-0.000028000
O	1.583114000	-0.538937000	0.000045000
C	3.944231000	-0.820069000	-0.000031000
C	0.432456000	0.305725000	0.000083000
C	-0.733193000	-0.679228000	0.000008000
N	-2.120860000	-0.047362000	-0.000011000
C	-2.334128000	0.796488000	-1.232347000
C	-3.133261000	-1.167236000	-0.000092000
C	-2.334209000	0.796379000	1.232386000
H	3.896901000	-1.464118000	-0.881023000
H	3.897071000	-1.463929000	0.881111000
H	4.875460000	-0.258528000	-0.000174000
H	0.444217000	0.946645000	-0.884369000
H	0.444190000	0.946532000	0.884618000
H	-0.681097000	-1.315287000	0.884341000
H	-0.681041000	-1.315220000	-0.884371000
H	-2.140386000	0.187388000	-2.113894000
H	-3.366137000	1.143506000	-1.239996000
H	-1.662430000	1.650991000	-1.211267000
H	-2.988554000	-1.773963000	0.891975000
H	-4.132909000	-0.736119000	-0.000115000
H	-2.988487000	-1.773896000	-0.892194000
H	-2.140541000	0.187196000	2.113892000
H	-1.662495000	1.650872000	1.211433000
H	-3.366213000	1.143410000	1.239989000

6. ACh^{+} (ctg)

Atom	x (Å)	y (Å)	z (Å)
C	-2.462625000	0.349899000	-0.012549000
O	-2.841143000	1.479107000	0.006393000
O	-1.085071000	0.158858000	-0.170171000
C	-3.323888000	-0.879129000	0.095714000
C	-0.458138000	-1.114046000	-0.079267000
C	0.959920000	-0.997107000	-0.622113000
N	1.860947000	0.062147000	0.022031000
C	3.289644000	-0.290804000	-0.310817000
C	1.567444000	1.439099000	-0.541412000
C	1.700484000	0.093843000	1.519251000
H	-3.016386000	-1.517408000	0.928113000
H	-3.274387000	-1.471439000	-0.822364000
H	-4.351507000	-0.559592000	0.252139000
H	-0.958564000	-1.865957000	-0.697661000
H	-0.464058000	-1.475350000	0.953879000
H	0.943432000	-0.764988000	-1.687196000
H	1.460056000	-1.957147000	-0.483042000
H	3.390890000	-0.372688000	-1.391947000
H	3.940834000	0.497925000	0.061888000
H	3.543561000	-1.238030000	0.162271000
H	0.528893000	1.686719000	-0.346152000
H	2.238766000	2.149492000	-0.061431000
H	1.754884000	1.418227000	-1.613855000
H	0.702828000	0.451101000	1.761932000
H	1.863100000	-0.906451000	1.918562000
H	2.443706000	0.776315000	1.928024000

7. ACh^+ (ctt)

Atom	x (Å)	y (Å)	z (Å)
C	2.886300000	-0.210489000	-0.000022000
O	3.713334000	-1.065773000	-0.000088000
O	1.551451000	-0.624087000	0.000108000
C	3.150277000	1.273653000	-0.000063000
C	0.464293000	0.289276000	0.000206000
C	-0.738514000	-0.658740000	0.000381000
N	-2.117987000	-0.012285000	-0.000023000
C	-2.323675000	0.830590000	-1.233240000
C	-3.140418000	-1.124791000	0.000618000
C	-2.323701000	0.832076000	1.232165000
H	2.725347000	1.753338000	0.885835000
H	2.724945000	1.753384000	-0.885743000
H	4.227015000	1.425725000	-0.000298000
H	0.480765000	0.924264000	-0.890873000
H	0.480962000	0.924302000	0.891252000
H	-0.689879000	-1.295425000	0.884012000
H	-0.689640000	-1.296025000	-0.882803000
H	-2.138403000	0.217332000	-2.113730000
H	-3.351210000	1.190698000	-1.241093000
H	-1.641831000	1.677333000	-1.214897000
H	-3.000109000	-1.732074000	0.892963000
H	-4.136299000	-0.684948000	0.000164000
H	-2.999856000	-1.733352000	-0.890816000
H	-2.138035000	0.220003000	2.113398000
H	-1.642150000	1.679029000	1.212566000
H	-3.351357000	1.191847000	1.239778000

8. Ch^+ (tg)

Atom	x (Å)	y (Å)	z (Å)
H	-3.101868000	0.676674000	0.154968000
O	-2.163395000	0.619975000	-0.056298000
C	-1.726316000	-0.727930000	0.009420000
C	-0.343974000	-0.845759000	-0.613326000
N	0.763537000	0.016050000	0.003552000
C	2.088264000	-0.543504000	-0.450680000
C	0.658822000	1.448556000	-0.475785000
C	0.710095000	-0.008976000	1.507956000
H	-2.370720000	-1.394169000	-0.576597000
H	-1.727116000	-1.097924000	1.041193000
H	-0.375148000	-0.576693000	-1.669606000
H	-0.013374000	-1.881971000	-0.526089000
H	2.101224000	-0.580482000	-1.538887000
H	2.884794000	0.107047000	-0.093477000
H	2.213159000	-1.544602000	-0.041033000
H	-0.314391000	1.839272000	-0.196345000
H	1.462538000	2.020445000	-0.014324000
H	0.769970000	1.459327000	-1.558813000
H	-0.198763000	0.487601000	1.838673000
H	0.730494000	-1.042858000	1.849811000
H	1.580257000	0.521868000	1.890537000

9. Ch^+ (tt)

Atom	x (Å)	y (Å)	z (Å)
H	3.625196000	0.156922000	-0.001339000
O	2.827991000	-0.383859000	0.000588000
C	1.678000000	0.439008000	0.000013000
C	0.537848000	-0.581261000	-0.010641000
N	-0.877571000	-0.021441000	-0.000017000
C	-1.831616000	-1.191622000	-0.027646000
C	-1.135158000	0.781274000	1.250525000
C	-1.131547000	0.837286000	-1.213413000
H	1.646579000	1.081778000	-0.887673000
H	1.639435000	1.072623000	0.894095000
H	0.628493000	-1.224973000	0.864941000
H	0.624225000	-1.202345000	-0.902829000
H	-1.652787000	-1.812764000	0.848096000
H	-2.852119000	-0.812682000	-0.015485000
H	-1.657151000	-1.767233000	-0.934817000
H	-0.501375000	1.664475000	1.253917000
H	-2.181579000	1.082086000	1.261306000
H	-0.917720000	0.160157000	2.117979000
H	-0.506413000	1.725591000	-1.168431000
H	-0.900237000	0.260417000	-2.107441000
H	-2.180635000	1.128800000	-1.219926000

10. AA

Atom	x (Å)	y (Å)	z (Å)
C	-0.089265000	0.128039000	0.000001000
C	1.393424000	-0.124225000	0.000000000
H	1.924615000	0.824687000	0.000029000
H	1.669893000	-0.708896000	0.880379000
H	1.669898000	-0.708842000	-0.880414000
O	-0.627558000	1.205993000	0.000000000
O	-0.792086000	-1.034775000	0.000000000
H	-1.732204000	-0.799580000	-0.000003000

11. H_2O

Atom	x (Å)	y (Å)	z (Å)
O	0.000000000	0.000000000	0.117076000
H	0.000000000	0.763464000	-0.468305000
H	0.000000000	-0.763464000	-0.468305000

Cartesian Coordinate of the Transition State Structure

1. TS-a

Atom	x (Å)	y (Å)	z (Å)
C	-2.651799000	0.183376000	-0.348397000
O	-2.816414000	-0.466933000	-1.299865000
O	-0.723724000	-0.177538000	0.388138000
N	2.284229000	0.114144000	0.016411000
C	1.264752000	-0.379693000	-1.018510000
C	2.497816000	-0.906755000	1.100080000
C	3.590547000	0.362527000	-0.688371000
C	1.812462000	1.407118000	0.640693000
C	-0.004501000	-1.020774000	-0.453018000
C	-2.837186000	1.594349000	0.056604000
H	1.022005000	0.492364000	-1.627473000
H	1.790817000	-1.106264000	-1.641599000
H	2.770299000	-1.858086000	0.645395000
H	3.304685000	-0.561230000	1.744736000
H	1.580560000	-1.007424000	1.674170000
H	3.420017000	1.048704000	-1.516411000
H	4.294693000	0.801233000	0.017280000
H	3.979795000	-0.582579000	-1.063667000
H	1.765306000	2.167263000	-0.137739000
H	0.823839000	1.233102000	1.057662000
H	2.531209000	1.702995000	1.404025000
H	-0.592983000	-1.306244000	-1.344260000
H	0.253206000	-1.969329000	0.041880000
H	-2.442078000	2.230305000	-0.736788000
H	-2.347428000	1.803802000	1.002153000
H	-3.912296000	1.769559000	0.155676000
O	-2.863165000	-0.704531000	1.203229000
H	-1.799044000	-0.729894000	1.134290000
H	-3.238328000	-1.576149000	1.009297000

2. TS-b

Atom	x (Å)	y (Å)	z (Å)
C	2.679539000	0.390504000	0.309093000
O	2.371184000	0.505095000	1.432595000
O	1.172261000	-0.516061000	-0.728017000
N	-2.468734000	0.087072000	-0.003559000
C	-1.073099000	0.137621000	-0.632883000
C	-2.431571000	0.566508000	1.424079000
C	-3.370813000	0.996621000	-0.797316000
C	-3.028889000	-1.310204000	-0.051579000
C	0.024607000	-0.710721000	0.029308000
C	3.237782000	1.271230000	-0.744710000
H	-1.191166000	-0.176981000	-1.670236000
H	-0.773822000	1.186408000	-0.622204000
H	-1.991886000	1.562193000	1.449146000
H	-3.450082000	0.599004000	1.808470000
H	-1.833892000	-0.117518000	2.020788000
H	-3.396476000	0.654246000	-1.830305000
H	-4.371690000	0.965653000	-0.368937000
H	-2.977709000	2.010689000	-0.752262000
H	-3.014700000	-1.659441000	-1.082718000
H	-2.424983000	-1.964586000	0.571420000
H	-4.052219000	-1.289430000	0.320274000
H	0.182617000	-0.395279000	1.075117000
H	-0.265549000	-1.771067000	0.046519000
H	2.701139000	2.220474000	-0.717500000
H	3.155570000	0.813862000	-1.725968000
H	4.290740000	1.447128000	-0.507793000
O	3.301510000	-1.204798000	-0.127966000
H	2.289571000	-1.301140000	-0.503029000
H	3.420696000	-1.716962000	0.685290000

3. TS-c

Atom	x (Å)	y (Å)	z (Å)
C	-2.642482000	0.245481000	0.434307000
O	-2.619150000	1.236903000	1.029690000
O	-0.698499000	-0.016133000	-0.351563000
N	2.273183000	0.090578000	0.066594000
C	1.511436000	-0.966458000	-0.742889000
C	1.990702000	-0.041111000	1.538271000
C	3.746077000	-0.102753000	-0.173042000
C	1.885480000	1.480026000	-0.386615000
C	0.047872000	-1.187336000	-0.338714000
C	-3.144261000	-1.135669000	0.595171000
H	1.582895000	-0.644061000	-1.782569000
H	2.064716000	-1.901083000	-0.626668000
H	2.209109000	-1.060357000	1.853765000
H	2.632272000	0.656099000	2.075103000
H	0.945814000	0.201442000	1.714286000
H	3.936508000	-0.070940000	-1.244656000
H	4.292135000	0.698344000	0.323254000
H	4.051672000	-1.066867000	0.230257000
H	2.174844000	1.594678000	-1.430203000
H	0.808341000	1.573403000	-0.277837000
H	2.420733000	2.202645000	0.228332000
H	-0.315444000	-1.949545000	-1.050875000
H	0.022455000	-1.670449000	0.651204000
H	-2.790389000	-1.517520000	1.554600000
H	-2.834536000	-1.780288000	-0.220650000
H	-4.236697000	-1.081026000	0.616932000
O	-2.756901000	0.489867000	-1.394820000
H	-1.725320000	0.317498000	-1.328112000
H	-2.923930000	1.425805000	-1.577714000

4. TS-d

Atom	x (Å)	y (Å)	z (Å)
C	-2.998464000	0.283423000	-0.367414000
O	-3.202203000	0.167284000	-1.500830000
O	-1.106618000	-0.521894000	0.031576000
N	2.610238000	0.022463000	-0.060085000
C	1.177737000	-0.418654000	-0.391052000
C	3.043355000	-0.521873000	1.274886000
C	3.524859000	-0.524802000	-1.124965000
C	2.718694000	1.523168000	-0.052198000
C	0.045846000	0.037264000	0.551196000
C	-3.133630000	1.326618000	0.673770000
H	0.968738000	-0.051822000	-1.396307000
H	1.193430000	-1.508542000	-0.417683000
H	2.931589000	-1.604779000	1.265556000
H	4.087027000	-0.257364000	1.439726000
H	2.425737000	-0.091630000	2.058705000
H	3.218968000	-0.128280000	-2.091425000
H	4.547910000	-0.222815000	-0.904021000
H	3.449484000	-1.610672000	-1.130062000
H	2.364606000	1.906255000	-1.007911000
H	2.114075000	1.925234000	0.756591000
H	3.761917000	1.799779000	0.094814000
H	0.246641000	-0.294521000	1.583255000
H	-0.004047000	1.139125000	0.573196000
H	-2.638091000	2.230458000	0.314846000
H	-2.721134000	1.006540000	1.624586000
H	-4.201360000	1.533620000	0.790458000
O	-3.251283000	-1.261776000	0.585936000
H	-2.205213000	-1.295898000	0.566804000
H	-3.601481000	-1.931706000	-0.019326000
